# The analysis of ascending aortic dilatation in patients with a bicuspid aortic valve using the ratio of the diameters of the ascending and descending aorta

**DOI:** 10.1186/1749-8090-9-108

**Published:** 2014-06-19

**Authors:** Yuki Nakamura, Masahiro Ryugo, Fumiaki Shikata, Masahiro Okura, Toru Okamura, Takumi Yasugi, Hironori Izutani

**Affiliations:** 1 Department of Cardiovascular and Thoracic Surgery, Ehime University Graduate School of Medicine, Shitsukawa, Toon City, Ehime 791-0295, Japan

**Keywords:** Bicuspid aortic valve, Aortic dilatation, Aortic valve replacement

## Abstract

**Background:**

A bicuspid aortic valve (BAV) is associated with premature valve dysfunction and abnormalities of the ascending aorta. The aim of our study was to assess the degree of ascending aortic dilatation by measuring the ratio of the dimension of the AAo to that of the descending aorta (DAo) using preoperative computerized tomography (CT).

**Methods:**

A review of our institutional clinical database identified 76 patients undergoing aortic valve replacement (AVR) and 73 control patients undergoing off-pump coronary artery bypass (OPCAB group) between September 2009 and April 2012.

**Results:**

There were 17 patients diagnosed with BAV (BAV group), and the remaining 59 patients had a tricuspid aortic valve (TAV group). The ratios of the dimensions of the AAo to that of the DAo (AAo/DAo) for each group were: BAV, 1.58 ± 0.25; TAV, 1.32 ± 0.11; and OPCAB, 1.29 ± 0.12. Interestingly, the AAo/DAo of the BAV group was significantly larger than that of the other groups.

**Conclusions:**

Although progressive AAo dilatation for BAV is well documented, the diameter of the AAo is currently the only estimate of aortic dilatation. In this study, we report that the ratio of the AAo and DAo diameters in patients with BAV can be a new index for assessing the dilatation of the AAo and differentiating the patients with BAV from those with TAV.

## Background

A bicuspid aortic valve (BAV) is one of the most common congenital heart diseases occurring in 1-2% of the general population. BAV is widely recognized as a frequent cause of aortic stenosis (AS) and/or aortic regurgitation (AR). BAVs are also associated with clinically serious abnormalities of the ascending aorta (AAo), including aortic dilatation and aortic dissection [1]. Although serial aortic size measurements are corrected for body surface area (BSA), as previously reported [2], operative aortic interventions for BAV are only considered, when the AAo is more than 4.5 cm in diameter [3-5]. We studied the degree of aortic dilatation in patients with a BAV using the ratio of the dimension of the AAo to that of the descending aorta (DAo), both of which were measured by preoperative computerized tomography (CT).

## Methods

**Figure 1:**
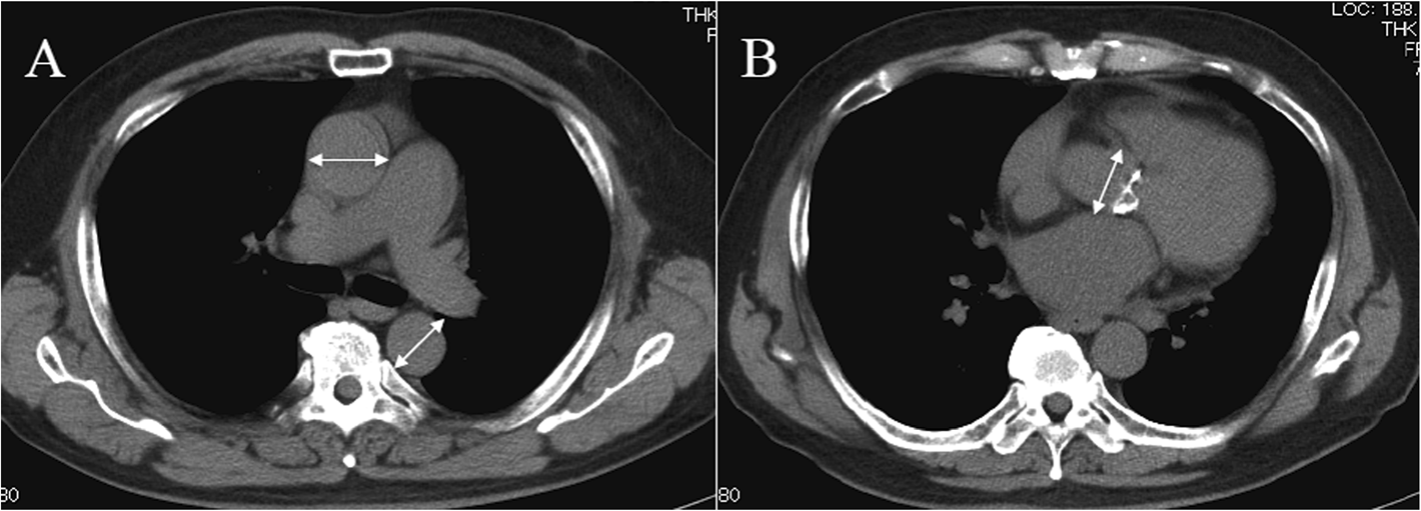
**Measurements of the aortic dimension. A**. The measurements of the diameter of the ascending aorta (AAo) were obtained from the largest diameter in the transverse plane of preoperative CT and the diameter of the descending aorta (DAo) was obtained from the same CT slice. **B**. The measurements of the aortic root (AoR) were obtained from the diameter of the sinus of Valsalva.

The study was approved by the institutional medical ethics committee and was consistent with the spirit of the Declaration of Helsinki. We reviewed the Ehime University Hospital computerized database and identified 87 consecutive patients with AS or AR undergoing aortic valve replacement (AVR) or aortic valve repair between September 2009 and April 2012. Patients with defined connective tissue disorders (for example Marfan syndrome or Ehlers-Danlos syndrome) were not included. We also excluded 8 patients whose preoperative CT scans were inappropriate, 2 patients with prosthetic aortic valve dysfunction undergoing redo AVR and a patient with dissecting aortic aneurysm of the descending aorta. Of this study, 17 patients were found to have a BAV from the operative records (BAV group), remaining 59 patients having a tricuspid aortic valve as TAV group. In this study, the replacement of the AAo, in addition to AVR, are performed when the AAo is more than 4.5 cm in BAV and TAV groups. There were 73 patients underwent off-pump coronary artery bypass (OPCAB) during the same period (OPCAB group). The aortic diameters were obtained for all patients by measurements in transverse planes of preoperative CT scans. We measured the largest diameters of the AAo and the diameter of the DAo from the same CT slice. The diameter of the aortic root (AoR) was also measured at the level of the Valsalva sinus (Figure 1). The ratio of the diameter of the AAo to that of the DAo (AAo/DAo) was obtained by dividing the dimension of the AAo by that of the DAo. The ratio of the diameter of the AoR to that of the DAo (AoR/DAo) was obtained in a similar fashion.

Continuous and categorical variables were compared among three groups. Kolmogorov-Smirnov’s goodness of fit test was used to assess the normality of each group and each variable. In this study, we confirmed the normality of each group. Differences were assessed using one-way analysis of variance. A probability value (p) < 0.05 was considered significant. We also assessed the correlation between the dimensions of the aorta and BSA, using the Pearson product–moment correlation coefficient.

## Results

**Figure 2:**
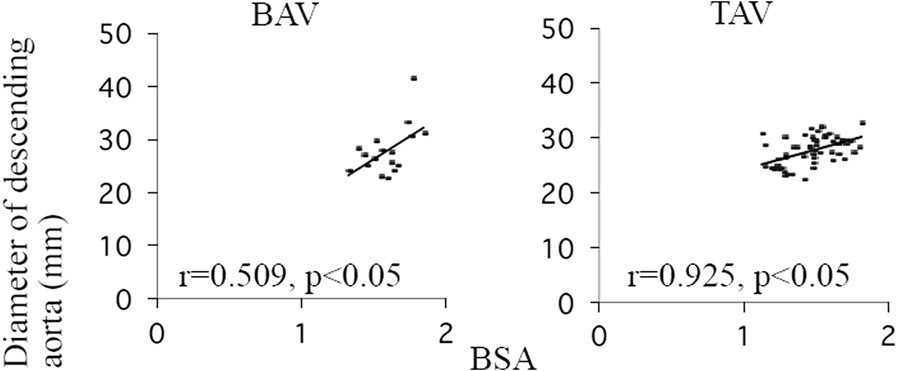
**Correlation between BSA and the dimensions of descending aorta in BAV and TAV groups.***BSA,* body surface area; *BAV,* bicuspid aortic valve; *TAV,* tricuspid aortic valve; r, correlation coefficient.

**Figure 3:**
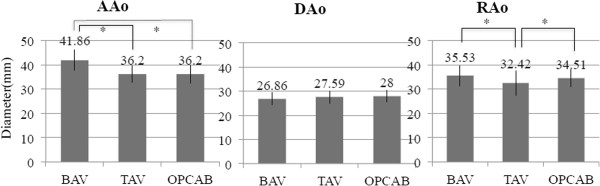
**Comparison of the diameters of the AAo, Dao and AoR among each group.***AAo*, ascending aorta; *DAo*, descending aorta; *AoR*, aortic root; *BAV*, bicuspid aortic valve; *TAV*, tricuspid aortic valve; *OPCAB*, off-pump coronary artery bypass. Values are mean ± SD; *, p < 0.05.

Baseline characteristics of the patients in the three groups are listed in Tables 1 and 2. The OPCAB patients had a relatively higher risk of arteriosclerosis than the other patients. The OPCAB patients weren’t found any significant aortic valve dysfunction and BAVs in preoperative echocardiography. Four patients in the BAV group underwent graft replacement of the ascending aorta in contrast to one patient in the TAV group. Three patients in the BAV group had AS and true ascending aorta dilatation and remaining 1 patient had AR and true ascending aortic dilatation. There was no poststenotic localized dilatation in patients with BAV. TAV patients more frequently had coronary artery disease than those in the BAV group. In the BAV and TAV groups, there was a positive correlation between BSA and the dimension of the DAo, which was assumed to be the normal size of the aorta (BAV r = 0.925, TAV r = 0.509) (Figure 2). There was a significant difference in the AAo diameter between the BAV and the TAV groups, as well as between the BAV and the OPCAB groups (p < 0.05). However, there was no significant difference in the AAo diameter between the TAV and the OPCAB groups. No difference in DAo diameter existed among the three groups. The statistically significant differences in the AoR size were seen between the BAV and the TAV groups (p < 0.05) (Table 3 and Figure 3).

**Table 1:** Baseline characteristics of patients in the three study groups

	**BAV (n = 17)**	**TAV (n = 59)**	**OPCAB (n = 73)**
Age	70 ± 7	77 ± 7	68 ± 9
Sex	13 (76.5%)	31 (52.5%)	62 (84.9%)
BSA	1.48 ± 0.40	1.46 ± 0.18	1.65 ± 0.17
Smoking	6 (35.3%)	24 (40.7%)	47 (64.4%)
Hypertension	10 (58.8%)	47 (79.4%)	54 (74.0%)
Hyperlipidemia	6 (35.3%)	20 (33.9%)	46 (63.0%)
Diabetes	3 (17.6%)	12 (20.3%)	48 (65.8%)
Chronic kidney disease	6 (35.3%)	20 (33.9%)	17 (23.3%)

*BSA,* body surface area.

*BAV,* bicuspid aortic valve.

*TAV,* tricuspid aortic valve.

*OPCAB,* off-pomp coronary artery bypass.

Values expressed as mean ± SD, or number (percent).

**Table 2:** Preoperative diagnosis of the patients enrolled in this study

	**BAV (n = 17)**	**TAV (n = 59)**
Aortic stenosis	15	46
Aortic regurgitation	2	13
Thoracic aortic aneurysm	4	1
Mitral regurgitation	2	3
Mitral stenosis	3	5
Tricuspid valve regurgitation	5	11
Atrial fibrillation	6	8
coronary artery disease	2	21

The number of patients in the BAV and TAV groups with aortic valve disease and other coexisting cardiovascular diseases, for which surgical procedures were added.

Values are expressed as number of patients.

**Table 3:** Aortic measurements for each of the study groups

	**BAV (n = 17)**	**TAV (n = 59)**	**OPCAB (n = 73)**
AAo (mm)	41.86 ± 4.21	36.20 ± 3.38	36.20 ± 3.74
DAo (mm)	26.86 ± 2.61	27.59 ± 2.61	28.00 ± 2.33
AoR (mm)	35.53 ± 4.20	32.42 ± 5.08	34.51 ± 3.55
AAo/DAo (ratio)	1.58 ± 0.25	1.32 ± 0.11	1.29 ± 0.12
AoR/DAo (ratio)	1.32 ± 0.16	1.19 ± 0.19	1.24 ± 0.11

Measurements were obtained from the transverse plane of preoperative CT scans as demonstrated in Figure 1.

*AAo* ascending aorta, *DAo* descending aorta, *AoR* aortic root.

Values represent mean ± SD.

**Figure 4:**
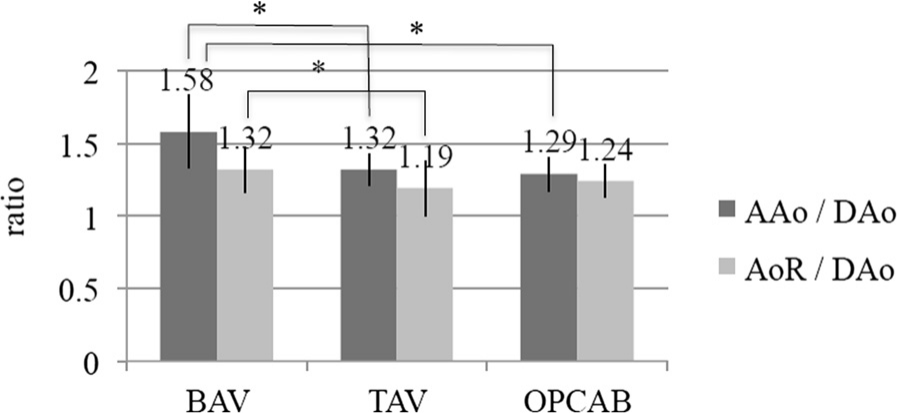
**Comparison of the aortic ratios (AAo/Dao and AoR/DAo) among each study group.***AAo*, ascending aorta; *AoR*, aortic root; *BAV*, bicuspid aortic valve; *DAo*, descending aorta; *OPCAB*, off-pump coronary artery bypass; *TAV*, tricuspid aortic valve. Values are mean ± SD; *, p < 0.05.

The AAo/DAo ratios in the three study groups were 1.58 ± 0.25 (BAV), 1.32 ± 0.11 (TAV), and 1.29 ± 0.12 (OPCAB), respectively. This ratio in the BAV groups was statistically larger than that of the other groups (p < 0.05). The AoR/DAo ratios in the three groups were 1.32 ± 0.16 (BAV), 1.19 ± 0.19 (TAV), and 1.24 ± 0.11 (OPCAB), respectively. The ratios in the BAV and OPCAB groups were significantly larger than that in the TAV group, respectively (p < 0.05); however, the AoR/DAo ratios did not differ significantly between the BAV and OPCAB groups (Figure 4).

## Discussion

The results of this study indicated the presence of obvious dilatation of the AAo in patients with a BAV. In contrast, the size of the DAo with BAV was similar to those of the other two groups. No difference in the AAo diameter existed between the TAV and OPCAB groups. These findings suggest that BAV is responsible for the dilatation of the AAo as reported in the prior studies [1,6-8]. Surgical aortic interventions for BAVs are generally recommended when the AAo is more than 4.5 cm in diameter [3-5]. However, it is clinically controversial whether BAV patients with a mildly dilated AAo should undergo an aortic procedure in addition to AVR. Aortic diameter is currently the only index for estimating dilatation of the AAo and alternative indexes are rarely discussed. As mentioned previously, considering that serial aortic size measurements are corrected for BSA [2], there is a possibility of misjudging the degree of AAo dilatation when only the size of AAo is measured. The results of our study demonstrated that the ratio of AAo/DAo ratios might be a new index for the degree of AAo dilatation, because this new index is less affected by individual differences in the patient’s body size or normal aortic size.

According to our data in the BAV group, some patients, who have AAo diameters of less than 4.5 cm, did indeed have relatively higher AAo/DAo ratios. Dvies et al. concluded that BAV patients had a higher rate of aortic growth than TAV patients [9] and there is increasing evidence that an aortic dilatation in patients with BAV occurs irrespective of valve function [6,7,10]. In consideration of these studies, these patients who are indicated for an AVR procedure might need an additional surgical aortic intervention. Because ascending aortic dilatation may progress even after successful AVR [11], close follow-up care is prudent for BAV patients with high AAo/DAo ratios after isolated AVR. Furthermore, our data indicated that this index is also useful for predicting the existence of BAV when the preoperative diagnosis of BAV by echocardiography or magnetic resonance imaging is uncertain.

Several limitations of our study should be acknowledged. First, the prognosis of BAV patients who have mild ascending aortic dilatation with relatively higher values of AAo/DAo cannot be determined from our study. Nevertheless, the degree of AAo dilatation obtained from this new index was obviously higher in BAV patients, and higher AAo/DAo ratios may indicate a risk for aortic complications. Further studies on the time course of aortic dilatation in patients with BAV are warranted. Second, each group had a relatively small sample size, particularly those including patients with BAV. As a matter of course, our sample sizes in this study were sufficient for statistical analysis, but this fact inevitably limits the statistical power of our analysis. Therefore, we must accumulate further cases to validate the findings of this study. Third, we cannot examine the histological differences of aortic valve and AAo between the BAV and TAV groups. Because of these limitations, we haven’t established the indication of replacing AAo using the new index yet. Nonetheless we suspect that we can assess AAo dilatation more accurately by using not only the AAo diameter but also AAo/DAo ratio. We believe that this new index will play a complementary role of the current indication for replacing the ascending aorta in patients with BAV in the future.

## Conclusion

Our study findings suggest that dilatation of the AAo in the patients with BAV was found more frequently than in the patients with TAV. This study strongly suggests that the novel AAo/DAo index obtained by CT scans is effective for differentiating the patients with BAV from those with TAV when overlooking BAV by usual echocardiographic study.

## Abbreviations

BAV: Bicuspid aortic valve; TAV: Tricuspid aortic valve; AAo: Ascending aorta; DAo: Descending aorta; AoR: Aortic root; CT: Computerized tomography; AVR: Aortic valve replacement; OPCAB: Off-pump coronary artery bypass; AS: Aortic stenosis; AR: Aortic regurgitation; BSA: Body surface area.

## Competing interests

The authors declare that they have no competing interests.

## Authors’ contributions

HI conceived of the study, and participated in its design and coordination and helped to draft the manuscript. FM, MR, MO, TO and TY participated in the design of the study. All authors read and approved the final manuscript.
